# Sentiment Analysis Using a Large Language Model–Based Approach to Detect Opioids Mixed With Other Substances Via Social Media: Method Development and Validation

**DOI:** 10.2196/70525

**Published:** 2025-06-19

**Authors:** Muhammad Ahmad, Ildar Batyrshin, Grigori Sidorov

**Affiliations:** 1 Centro de Investigación en Computación, Instituto Politécnico Nacional, Mexico City, 07738, Mexico, 52 5591887293

**Keywords:** opioid overdose, deep learning, large language models, high dose, NLP, chronic pain, BERT, social media, suicide, ChatGPT, natural language processing, bidirectional encoder representations from transformers, data mining, Reddit

## Abstract

**Background:**

The opioid crisis poses a significant health challenge in the United States, with increasing overdoses and death rates due to opioids mixed with other illicit substances. Various strategies have been developed by federal and local governments and health organizations to address this crisis. One of the most significant objectives is to understand the epidemic through better health surveillance, and machine learning techniques can support this by identifying opioid users at risk of overdose through the analysis of social media data, as many individuals may avoid direct testing but still share their experiences online.

**Objective:**

In this study, we take advantage of recent developments in machine learning that allow for insights into patterns of opioid use and potential risk factors in a less invasive manner using self-reported information available on social platforms.

**Methods:**

This study used YouTube comments posted between December 2020 and March 2024, in which individuals shared their self-reported experiences of opioid drugs mixed with other substances. We manually annotated our dataset into multiclass categories, capturing both the positive effects of opioid use, such as pain relief, euphoria, and relaxation, and negative experiences, including nausea, sadness, and respiratory depression, to provide a comprehensive understanding of the multifaceted impact of opioids. By analyzing this sentiment, we used 4 state-of-the-art machine learning models, 2 deep learning models, 3 transformer models, and 1 large language model (GPT-3.5 Turbo) to predict overdose risks to improve health care response and intervention strategies.

**Results:**

Our proposed methodology (GPT-3.5 Turbo) was highly precise and accurate, helping to automatically identify sentiment based on the adverse effects of opioid drug combinations and high-risk drug use in YouTube comments. Our proposed methodology demonstrated the highest achievable *F*_1_-score of 0.95 and a 3.26% performance improvement over traditional machine learning models such as extreme gradient boosting, which demonstrated an *F*_1_-score of 0.92.

**Conclusions:**

This study demonstrates the potential of leveraging machine learning and large language models, such as GPT-3.5 Turbo, to analyze public sentiment surrounding opioid use and its associated risks. By using YouTube comments as a rich source of self-reported data, the study provides valuable insights into both the positive and negative effects of opioids, particularly when mixed with other substances. The proposed methodology significantly outperformed traditional models, contributing to more accurate predictions of overdose risks and enhancing health care responses to the opioid crisis.

## Introduction

### Background

Opioid overdose occurs when someone takes an excessive amount of prescribed or illicit drugs, such as heroin or fentanyl, which can cause potentially life-threatening symptoms by interacting with receptors in the brain and nervous system to reduce pain. Chronic pain, one of the leading causes of disability and overall disease burden worldwide [1,2], is a significant factor in the increasing use of opioid drugs mixed with illicit substances, with an estimated 20%-30% of the global population experiencing chronic pain [3,4]. The annual economic impact of chronic pain ranges from US $560 to $635 billion in the United States [5-7]. The challenging nature of chronic pain makes it one of the most persistent medical issues, presenting various diagnostic and treatment difficulties [8]. In the pharmacological management of chronic pain, opioids have long been considered essential medications for patients. Although their effectiveness in treating serious pain is generally accepted, the use of opioids for chronic pain remains controversial due to long-term side effects such as tolerance and dependence [9,10]. These issues, along with prescription and misuse, have contributed to a significant global health crisis known as the opioid crisis [11], which has resulted in approximately 500,000 overdose deaths in the United States, with nearly 70,000 fatalities reported in 2020 alone [12].

In recent years, sentiment analysis has attracted exponential interest from researchers. The growing number of scientific publications, forums, and related conferences highlights its potential for future development. Social media platforms such as X (formerly known as Twitter), Facebook, Instagram, Reddit, and YouTube play a key role in this expansion, with over 58% of the world’s population actively sharing their opinions, experiences, and concerns on these platforms [13]. These platforms provide researchers with valuable insights into health determinants by allowing the analysis of lifestyle choices, habits, and personal experiences. Social media’s role in medical research is profound as it enables real-time global observations of important clinical topics, including influenza spread, suicide risk factors, and substance use trends [14-19].

Recent advances in natural language processing (NLP) have facilitated large-scale social media data analysis, making significant contributions to fields such as suicide risk detection, adverse drug reaction identification, and misinformation classification [20-22]. However, there remains a notable gap in applying key phrase extraction techniques to self-reported health-related content on social media, particularly within online health communities. The rise of web-based health care platforms has propelled automatic sentiment analysis of medical reviews into a new era of data-driven insights. This method allows researchers to analyze vast amounts of web-based user-generated data, uncovering hidden patterns about the side effects of opioid drugs. These insights are crucial for refining pharmacovigilance programs, ensuring drug safety and effectiveness. Over time, sentiment analysis in NLP has evolved significantly, enabling more accurate and meaningful interpretations of user experiences with medicines [23,24].

### Prior Work

Recent years have witnessed the trend of studying opioid use disorders using social media data such as YouTube comments, X, and Instagram. Social media platforms have become essential for analyzing user-reported experiences with opioid drugs, particularly when mixed with illicit substances, as they offer valuable insights into drug use behaviors and potential overdose risks.

Carabot et al [25] used state-of-the-art machine learning (ML) models on Twitter posts related to opioid drugs. They collected a dataset from January 1, 2019, to December 31, 2020, focusing on user experiences and perceptions of these drugs. They gathered a total of 256,218 Twitter posts. They used preprocessing techniques, and only 27% of the tweets were filtered out, which shows relevancy; after preprocessing, they conducted a manual analysis of 7000 tweets using a detailed codebook. They classified users as patients, health care professionals, or institutions and distinguished between medical and nonmedical content. The findings showed that fentanyl was the most discussed opioid, with patients dominating the conversation, while health care professionals’ tweets garnered the most engagement.

Swaileh et al [26] explored sentiment analysis in NLP to improve the understanding of public health and medication experiences. They used a hybrid model that combined traditional methods with advanced ML. Their proposed methodology achieved a high accuracy of 99% in sentiment classification. Their goal was to improve pharmacovigilance and inform public health initiatives by analyzing user feedback on health care and medications.

Chenworth et al [27] conducted a study to analyze public perceptions of methadone and buprenorphine-naloxone (Suboxone) through Twitter posts. They performed manual and automatic analyses, identifying common themes such as access, stigma, and treatment, with limited positive sentiment about the medications. Despite their proven effectiveness, the study suggests that public perceptions may contribute to the underutilization of these treatments for opioid use disorder.

Al-Hadhrami et al [28] explored the performance of deep learning (DL) techniques including bidirectional long short-term memory (BiLSTM) and a hybrid BiLSTM convolutional neural network (CNN) for sentiment analysis of drug-related reviews. They used Global Vectors for Word Representation (GloVe) word embedding methods and achieved an accuracy rate of 96%. The results underscore the enhanced performance of these models in analyzing patient sentiments, demonstrating the value of DL techniques in this context.

Chakrapani et al [29] discussed the challenge of analyzing the mindset of patients affected by acute diseases by introducing a framework that uses a sociomedical dataset of reviews and feedback. They used preprocessing techniques, n-gram tokenization, and polarity scoring to extract sentiments, followed by a probabilistic latent Dirichlet allocation model for review aggregation. They applied various ML models and evaluated the performance of the models in understanding patient perspectives.

Nair et al [30] focused on creating a drug review classification system to label user reviews into multiple classes, such as positive, negative, and neutral, by using publicly available datasets from drugs.com. They applied 3 variants of the pretrained bidirectional encoder representations from transformers (BERT) model, namely mBERT, SciBERT, and BioBERT, to generate embeddings used as features for various ML classifiers, including decision trees (DTs) and DL models. Model performance was assessed using precision, recall, and *F*_1_-score metrics.

Gandy et al [31] assessed the efficacy of 3 automated sentiment analysis tools—VADER, TEXT2DATA, and LIWC-22—against manually labeled datasets of YouTube comments related to opioid epidemics. The LIWC-22 model achieved the highest accuracy with an 88% *F*_1_-score, whereas VADER achieved 83%, and TEXT2DATA achieved 82%. The results suggest that these models can be effectively applied to social media analyses.

Although prior studies have used state-of-the-art ML and NLP models for opioid-related research, they often focused on basic sentiment classification such as positive and negative opinions and did not consider detailed discussions about mixed drug use. Many previous models did not include the many ways drugs can be mixed or their effects, which is essential for fully understanding opioid misuse. Unlike past studies, our research introduces a unique multiclass methodology with 6 different categories, including a mix of opioids and other substances. This classification captures the complexity of real-world drug use, which other studies may overlook. By using a large language model (LLM), we can better study and sort these mixed-drug experiences, detecting subtle feelings and trends that older models cannot. Our approach does more than just basic sentiment analysis. It overcomes the weaknesses of past models and gives a clearer, more complete picture of opioid misuse.

### Objective

This study aims to validate a methodology that uses YouTube video comments for sentiment analysis, focusing on instances where people discuss opioid drug use mixed with other substances, increasing the risk of overdose and adverse effects. By using advanced NLP techniques and LLMs such as GPT-3.5 Turbo, this research seeks to uncover hidden patterns and derive meaningful insights from discussions about drug use. Although the information shared on social media platforms can provide valuable insights into individual experiences, it is important to note that these platforms do not directly reflect the cause and usage situations in real-world settings. Despite the high penetration of social media, the data derived from these sources cannot always be used to determine the full context of opioid misuse, overdose, or adverse effects in the real world. Unlike traditional studies that focus solely on sentiment classification, our approach directly contributes to health care by identifying high-risk behaviors and potential opioid misuse patterns, such as the combination of opioids with other substances that significantly increase overdose risk. By analyzing both the emotional tone and detailed drug use experiences, our work aims to empower public health organizations with actionable intelligence to address emerging drug trends proactively and uncover risk factors linked to the misuse of opioids, including adverse physical effects and emotional responses, which could inform public health interventions. The use of ML, DL, and LLMs such as GPT-3.5 Turbo is critical for detecting subtle patterns within large amounts of social media data, which can be difficult to identify manually. Although social media platforms do not directly reflect the full context of opioid misuse or overdose situations in the real world, these advanced techniques enhance our ability to derive accurate and actionable insights from online discussions about opioid misuse, ultimately improving patient outcomes and informing intervention strategies. Although social media data cannot fully capture the complexities of real-world usage, these techniques enable the identification of emerging risks and behavioral trends that might otherwise go unnoticed. This approach facilitates faster responses to public health concerns, enhances community safety, and minimizes reliance on manual intervention by providing comprehensive, data-driven analyses.

To achieve these objectives, we developed a meticulously curated, multilabeled corpus, where each comment was manually annotated to reflect observed adverse effects related to opioid use. The dataset encompasses 6 distinct sentiment categories, including both positive experiences (eg, pain relief, euphoria, relaxation) and negative outcomes (eg, nausea, sadness, and respiratory depression). The selection of these 6 categories was driven by a need to capture the full spectrum of user experiences, both favorable and adverse, when discussing opioid use. By including both subjective emotional states and physical effects, we can gain a more comprehensive understanding of how different opioids impact individuals. This classification approach also supports the creation of precise, targeted interventions aimed at improving health outcomes, as it allows for the identification of both beneficial and harmful patterns in opioid usage.

### Contributions

This paper makes the following contributions to the literature.

We applied the schema to build a comprehensive dataset for sentiment analysis that contains opioid mixed with illicit drugs for health care professionals, accurately annotated with high-quality labels able to identify high-risk behaviors and develop targeted interventions.

We trained and tested an LLM (GPT-3.5 Turbo) on YouTube comments where people discuss using opioid drugs mixed with other substances that can cause death. This approach provides health care professionals and policymakers with real-time, data-driven insights into opioid use trends, enabling better response strategies and prevention measures.

We conducted a comprehensive series of experiments that demonstrated that the proposed methodology achieved the best performance compared to the baseline.

The proposed framework (GPT-3.5 Turbo) demonstrated an *F*_1_-score of 0.95 in multiclass to our dataset. This represents performance improvements of 3.26% in *F*_1_-score compared to the baseline model (extreme gradient boosting [XGBoost] demonstrated an *F*_1_-score of 0.92).

By bridging the gap between social media sentiment analysis and health care research, this study highlights how NLP-driven methodologies can contribute to public health strategies, improve patient safety, and enhance health care delivery. However, while NLP models can significantly assist in trend identification and risk assessment, human oversight remains crucial in interpreting results and implementing appropriate public health interventions.

## Methods

### Overview

**Figure 1.**
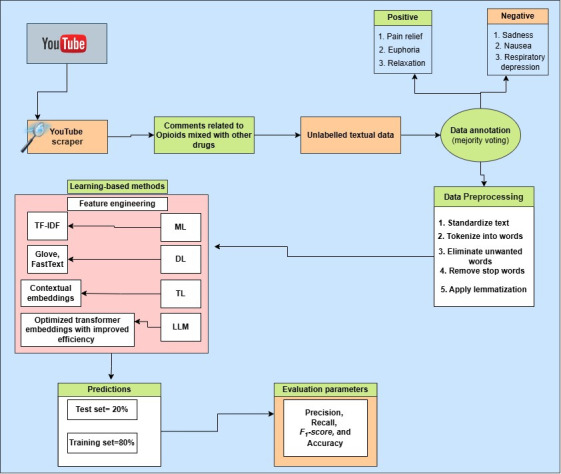
Architecture of proposed solution. DL: deep learning; LLM: large language model; ML: machine learning; TL: transfer learning.

This section outlines the methodologies used to create a robust sentiment analysis system. Initially, the research design is presented in a descriptive manner, with detailed explanations provided for each component in the flow diagram (Figure 1). The methodology includes multiple phases: (1) construction of dataset, (2) annotation guidelines, (3) annotation selection, (4) annotation agreement, (5) preprocessing and analysis of the data, (6) features extraction, and (7) application of models and training and testing.

### Construction of Dataset

This section outlines the construction of our dataset for sentiment analysis related to opioid overdose discussions on YouTube. First, we selected videos with more than 10 million views that were related to opioid overdose to ensure that the video had a sufficient number of comments discussing the mixing of opioid drugs with other substances. For inclusion, we selected videos based on their relevance to opioid misuse, focusing on videos with clear and significant discussions of opioid drugs mixed with other substances. We excluded videos with irrelevant content, off-topic discussions, or those lacking substantial user comments on opioid misuse and its adverse effects. For data selection, we chose videos from 2020 to 2024. One of the reasons for selecting YouTube comments from this time period was to capture recent discussions in which individuals shared their fresh experiences, especially during the COVID-19 pandemic when opioid misuse surged and individuals turned to social media more frequently to share their personal experiences. We used 20 different opioid-related keywords, such as “kratom,” “fentanyl,” “heroin,” “codeine,” and “buprenorphine,” to filter the relevant samples and drug occurrences and their adverse effects as reported by opioid users. Second, we prepared a code using the YouTube application programming interface in Python, which allowed us to collect approximately 300,000 comments from different videos that reflect self-reported and personal experiences shared by users. For this study, we selected only English-language videos and comments. Third, we manually categorized the dataset into 6 sentiment categories based on the adverse effects shared by the user, ensuring a more accurate and context-sensitive classification than traditional autoannotated methods. Unlike automatic annotation techniques, which often struggle to capture the complexity of user experiences, our manual categorization process allows for a deeper understanding of the nuanced nature of opioid use and its associated effects. By classifying the dataset into sentiment categories, we aim to develop a robust model capable of understanding both the sentiment of user concern and the adverse effects they report. This manual approach ensures high accuracy and precision, which is crucial for identifying patterns related to opioid misuse and overdose risks. An example structure of the dataset, showing sample entries and classifications, is presented in Table 1 (see annotation guideline section). Figure 1 illustrates the proposed methodology and design used in this study, highlighting the contributions of this more detailed, context-aware classification method.

**Table 1.** Samples from the dataset.

Comment text	Sentiment
I felt this amazing rush of happiness, like everything was perfect for a few hours. I know it’s risky, but nothing else makes me feel that alive.	Euphoria
The pain was unbearable, so I mixed a little extra with my regular dose. It worked for the pain, but I feel uneasy about it – I know it’s dangerous.	Pain relief
After mixing opioids with alcohol, I could barely breathe; it was like my chest was weighed down. Scariest experience of my life.	Respiratory depression
I thought it would help me forget, but all it did was make me feel numb and more alone. It’s not worth the spiral I’m in now	Sadness
Just a small dose with some weed, and I felt completely at ease, like I didn’t have a care in the world. It’s tempting to keep doing it, but I worry about the risks	Relaxation
I thought it would help me unwind, but instead, I felt so sick. I could barely keep anything down, and it just wasn’t worth it	Nausea

### Annotation Guidelines

After the collection of data, we accurately classified the samples related to opioid overdose drugs to gain insights into public sentiment. Each sample was labeled using predefined criteria, allowing us to classify based on the effects of drugs, including positive (pain relief, euphoria, relaxation) and negative experiences (nausea, sadness, and respiratory depression). Furthermore, the categorizations of posts are presented in Table 1 and the annotation rules are listed here:

1. Full comment reading: Mark only after reading the full comment carefully. Skim-reading will be not allowed.
2. Annotation consistency: Use accurate labels as defined in these guidelines. Any deviation, such as “Maybe” or “Unclear,” is not permitted.
3. Data quality check: Annotators must verify their annotated labels before finalizing as it is a necessary step to ensure accuracy and consistency.
4. Out-of-scope content: If a YouTube sample is off-topic, such as spam or irrelevant content, mark it as “Not applicable” and remove it from the corpus.
5. Pain relief: If a sample mentions opioids or mixing other substances with opioids providing relief from physical pain, including chronic pain or injury-related pain, label it as pain relief.
6. Euphoria: If a sample demonstrates a sense of joy, bliss, or intense well-being after using opioids or opioid mixtures, label it as euphoria.
7. Relaxation: If a sample mentions the relaxing, soothing, or sedative effects of opioids or opioids leading to relaxation from stress and anxiety, mark it as relaxation.
8. Nausea: Samples that indicate feeling sick or queasy or vomiting after using opioids or other drugs mixed with opioids should be marked as nausea.
9. Sadness: Samples that indicate feelings of hopelessness or emotional downers linked to opioid use or other mixtures with opioids are marked as sadness.
10. Respiratory depression: Samples that indicate difficulty breathing or a sense of being unable to breathe properly, often as a result of opioid use, should be marked as respiratory depression.

### Annotation Selection

Identifying sentiment analysis in multiclass was not an easy task; it presented significant challenges. Each of these classes added another layer of complexity, requiring annotators to carefully interpret and distinguish nuanced information within the text. This made it crucial to select annotators with strong analytical skills and attention to detail. To ensure high-quality labeling for our research, we carefully selected 5 students with strong backgrounds in annotation and ML. The selected candidates were postgraduate students in computer science. We assigned 300 comments to each candidate to label the dataset; separate Google sheets were created for individuals to record their work, which allowed us to track and evaluate their performance individually. After reviewing the results, 3 of the candidates consistently agreed on the same labels across most comments, demonstrating a high level of reliability and accuracy. Based on these results, these candidates were finalized for the full annotation of this dataset.

### Annotation Agreement

During the annotation process, variations in opinion arose among annotators. It is essential to analyze these inconsistencies effectively. This evaluation was carried out by calculating the interannotator agreement, which measures the quality and consistency of the annotation process. For our annotation procedure, we used the Fleiss κ statistic to determine this agreement. Fleiss κ is particularly useful when dealing with 3 or more annotators and categorical output labels. In our case, the value of κ was found to be 0.79, suggesting substantial agreement between annotators, as it falls within the range of 0.61 to 0.80. Table 2 provides the full interpretation of κ values.

**Table 2.** Interpretation of κ values for agreement between annotators.

κ value	Interpretation
<0	Less than chance agreement
0.10‐0.20	Slight agreement
0.21‐0.40	Fair agreement
0.41‐0.60	Moderate agreement
0.61‐0.80	Substantial agreement
0.81‐0.99	Almost perfect agreement

### Ethical Considerations

This study used secondary data comprising publicly available, user-generated content collected from Reddit to analyze public sentiment on opioids mixed with other substances. The data were obtained from existing, publicly accessible Reddit posts that do not contain any personally identifiable information. All content was anonymized and analyzed in aggregate to ensure the privacy and confidentiality of individuals.

There was no direct interaction with Reddit users, and no attempt was made to trace or reidentify individuals. Given that the study involved only the analysis of publicly available data, with no human subject intervention or collection of private or identifiable information, institutional review board approval was not required.

### Data Preprocessing

YouTube is a video-based social networking platform where video descriptions and comments often contain URLs, hashtags, emoticons, misspelled words, internet slang, and informal grammar expressions. In this context, data preprocessing is crucial to improving text quality, making it suitable for ML models and enhancing overall model performance, especially for sentiment analysis. For traditional ML models such as DTs and XGBoost, we applied standard preprocessing steps, including text normalization by converting all text to lowercase, removing extra spaces and newline characters, tokenizing the text into individual words, and filtering out nonalphanumeric characters. Additionally, stop words were removed using a predefined list, words shorter than 3 characters were discarded, and lemmatization was applied to ensure consistency by reducing words to their base forms. However, for DL models and transformer-based architectures such as GPT-3.5 Turbo, we avoided unnecessary preprocessing steps like tokenization, stop word removal, and term frequency-inverse document frequency (TF-IDF) transformations, as these models are designed to process raw text input directly using their own internal mechanisms for text representation. Instead, we only performed minimal cleaning (removing URLs, special characters, and excessive punctuation) to maintain linguistic integrity while reducing noise. This ensures that transformer-based models fully leverage their contextual embeddings, improving sentiment classification accuracy while preventing the loss of valuable textual information.

### Data Augmentation

To enhance the performance and robustness of our proposed models, we used the back translation technique for data augmentation. For the translation process, we used the Google Translate application programming interface, which offers broad language support and high-quality translations. To handle large volumes of text efficiently, we developed custom scripts that automated the translation process. After back translation, we conducted a manual quality check on a sample of the augmented data to ensure that the original meaning was retained and that no significant information was lost during the translation.

### Dataset Statistics

Multimedia Appendix 1 depicts a word cloud comprising keywords extracted from posts in the dataset related to the topic of opioid overdose. The word cloud visually highlights the most frequent terms, emphasizing the critical themes discussed in the dataset. Multimedia Appendix 2 illustrates the distribution of labels for each class used in the corpus for sentiment analysis. The chart visually represents the frequency of each sentiment class in the dataset. Multimedia Appendix 3 provides an overview of the text data’s structure. It shows that the dataset contained a total of 10,129,795 characters and had a vocabulary size of 31,893 unique words. On average, each sentence had 21.39 words, and each post contained 5.02 sentences. The average post length was 541.32 characters. Additionally, each word had an average of 4.86 characters. These values give a clear picture of the dataset’s complexity, showing how detailed and varied the posts are in terms of sentence and word length.

### Feature Extraction

After cleaning the text, the next step was feature extraction, where we converted text into numerical form for the ML models. In traditional ML, we used TF-IDF, as shown in Equations 1 and 2, which assigns importance to words based on their frequency in a document and rarity across the dataset. This helps highlight key terms for sentiment analysis. For DL, we used GloVe and FastText embeddings, as shown in Equations 3 and 4. GloVe creates fixed vector representations based on word co-occurrence in large text collections, capturing meaningful relationships between words. FastText improves upon this by considering subword information, which helps in understanding rare and misspelled words, making the model more robust. For transformer-based models and LLMs, we used pretrained embeddings from models like BERT and ChatGPT. These models capture deep contextual meanings by analyzing entire sentences rather than individual words. Unlike traditional methods, transformers dynamically understand context, improving sentiment analysis accuracy by recognizing complex language patterns.


(1) $$TF=\frac{Number\;of\;times\;term\;t\;appears\;in\;a\;document}{Total\;number\;of\;terms\;in\;the\;document}\;\;\;\;\;$$

The inverse document frequency of a term reflects the inverse proportion of documents that contain that term. Terms with technical jargon, for example, hold greater significance compared to words found in only a small percentage of all documents. The inverse document frequency can be computed using Equation 2:


(2) $$IDF=\frac{Number\;of\;documents\;in\;the\;corpus}{Number\;of\;documents\;in\;the\;corpus\;containing\;term}\;\;\;\;\;$$

TF-IDF can be calculated using Equation 3:


(3) TF−IDF =TF×IDF

FastText extends Word2Vec by representing words as bags of character n-grams. The embedding for a word *w* is calculated using Equation 4:


(4) $$Vw=\sum_{g\in G\left(w\right)}Vg\;$$

Where:

- A set of character n-grams in the word *w*.
- *Vg* is the vector representation of each n-gram *g*.

This allows FastText to generate embeddings for out-of-vocabulary words by combining the embeddings of their character n-grams.

GloVe creates word embeddings based on the co-occurrence matrix of words. Equation 5 is derived from the ratio of co-occurrence probabilities.


(5) $$Cost=\sum_{i,j}^{V}f\left(X_{i,j}\right){\left(V_{i}^{T}V_{j}+b_{i}+b_{j}-log\left(x_{i,j}\right)\right)}^{2}$$

Where:

- *X_i,j_* is the number of times word *j* occurs in the context of word *i*.
- *V* is the vocabulary size.
- *V_i_* and *V_j_* are the embeddings for words *i* and *j*.
- *b_i_* and *b_j_* are bias terms for the words.
- *f* (*X_i,j_*) is a weighting function to downweight the influence of very frequent words.

### Application of Models and Training and Testing

**Figure 2.**
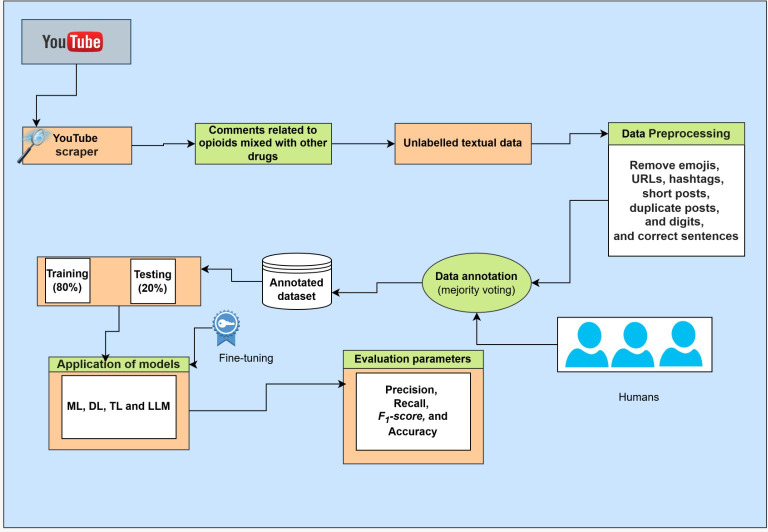
ML-, DL-, and LLM-based model training pipeline for multiclass text classification. DL: deep learning; LLM: large language model; ML: machine learning; TL: transfer learning.

In this section, we discuss the application of various models including ML models, DL models, transformer-based models, and LLMs such as GPT-3.5 Turbo. After feature extraction, the data were split into training and testing sets. The training set was processed to train ML algorithms including support vector machine (SVM), logistic regression (LR), k-nearest neighbor (KNN), and XGBoost, as well as 2 DL models (CNNand BiLSTM), 2 pretrained transformer models (BERT and GPT-2), and 1 LLM (GPT-3.5 Turbo). To accomplish this objective, we randomly partitioned the dataset into 80% for training and 20% for testing, as shown in Figure 2, which illustrates the ML-, DL-, and LLM-based model training pipeline for multiclass text classification. These approaches were evaluated using recall, precision, and *F*_1_-score to quantify the performance of the models. We calculated these metrics using the following equations.

Precision: The total number of correct predictions in our model was retrieved during document retrieval.

Recall: This indicates the classifier’s ability to identify all relevant instances in the dataset.

*F*_1_-score: The *F*_1_-score is a metric that combines precision and recall.

Equation 8 was used for *F*_1_-score, while Equations 6, 7, and 9 were used for precision, recall, and accuracy, respectively:


(6) $$Precision=\frac{TP}{FP+TP}$$

(7) $$Recall=\frac{TP}{FN+TP}$$

(8) $$F1-\;score=2\times \frac{\;Recall\;\times Precision}{Recall+\;Precision\;}$$

(9) $$Accuracy=\frac{TP+TN}{TP+TN+FP+FN}$$

Where TP is true positive, TN is true negative, FP is false positive, and FN is false negative.

## Results

### Overview

This section discusses the results derived from the methodology, implementation, and experiments outlined earlier. For ML models, we used GridSearchCV for hyperparameter optimization, testing parameters such as regularization (eg, C and gamma for SVM), penalty terms for LR, and boosting-related settings like learning rate, number of estimators, and maximum tree depth for XGBoost. For KNN, we tuned parameters like the number of neighbors and weight functions. In the case of DL models, adjustments were made to epochs, batch sizes, and learning rates to fine-tune the BiLSTM and CNN architectures for optimal performance. For transfer learning models, fine-tuning involved modifying pretrained weights and adapting hyperparameters such as learning rates, sequence lengths, and transformer-specific configurations to improve BERT and GPT-2 on the dataset. Each model’s performance was systematically optimized by fine-tuning its parameters to maximize its effectiveness. A comprehensive overview of the hyperparameters and grid search used in the proposed approach is provided in Table 3.

**Table 3.** Optimum values identified for the hyperparameters of each learning approach.

Learning approach and models		Hyperparameter	Fine tuning pipeline
Large language model: GPT-3.5 Turbo		Learning rate, epoch, batch size, seed	2, 3, 29, 1414121048
Transformer: bidirectional encoder representations from transformers, RoBERTa, crosslingual language model – RoBERTa		Learning rate, epoch, batch size, optimizer, loss function	2e-5, 3, 32, AdamW, CrossEntropyLoss
**Machine learning**			
	K-nearest neighbors	n_neighbors, weights	5, uniform
	Extreme gradient boosting	n_estimators, max_depth, learning_rate	100, 6, 0.3
	Decision tree	random_state, max_depth	42, 10
	Logistic regression	random_state, max_iter, C, solver	42, 1000, 0.1, liblinear
Deep learning: bidirectional long short-term memory and convolutional neural networks		learning rate, epoch, embedding_dim, batch size,	0.1, 3, 300, 32

### Software and Hardware

Experiments were conducted on a Lenovo laptop powered by an Intel Core i7, 8th generation processor with 4 cores, bus speed of 8 gigatransfers/second, 24 GB of RAM, and 1 TB of storage. The operating system used was Windows 10 Pro (Microsoft Corp), which provided a stable environment for development and execution. To perform the predictive analysis, Google Colab was selected for programming and easy access to a Python environment. We used Python version 3.12.4. The *Scikit-Learn* [32] package was used for ML models, while *TensorFlow* [33] and *Keras* [34] were used for DL tasks. For transformer-based models, the Hugging Face Transformers library was used. Model training was performed on an NVIDIA Tesla T4 GPU with 2560 CUDA cores and 16 GB GDDR6 memory.

### Results for ML

In this section, we will explore the performance of several traditional ML models applied to sentiment analysis, specifically focusing on the complex topics of opioid overdose and drug mixing with other substances. To tackle this, we used 6 models including LR, KNN, random forest, and SVM. Each model was evaluated to understand how well it can detect sentiment in this sensitive area, aiming to identify patterns and nuances within the data related to drug use.

Table 4 shows the performance metrics of 4 different ML models: LR, DT, KNN, and XGBoost. We used 4 different evaluation metrics to assess the performance of these models including precision, recall, *F*_1_-score, and accuracy. Among all models, XGBoost achieved the highest scores on all metrics (0.92 for all 4 metrics), demonstrating that it performs exceptionally well in making correct predictions in our sentiment analysis task. DT follows closely behind, with 0.87 across the board, showing strong performance just slightly lower than that of XGBoost. KNN also performed well, with an *F*_1_-score of 0.85, but LR, while decent, lagged behind with a score of 0.74 in all metrics, suggesting that it may not be a suitable choice for our sentiment analysis task. Overall, XGBoost was the clear winner in terms of accuracy and balanced performance.

**Table 4.** Results for machine learning models.

Model	Precision	Recall	*F*_1_-score	Accuracy
Logistic regression	0.74	0.74	0.74	0.74
Decision tree	0.87	0.87	0.87	0.87
K-nearest neighbors	0.85	0.86	0.85	0.86
Extreme gradient boosting	0.92	0.92	0.92	0.92

### Results for DL

In text classification tasks, choosing the right model and word embedding technique is essential for achieving accurate results. For this analysis, we compared the performance of 2 popular DL models (CNN and BiLSTM) using 2 different types of word embeddings: FastText and GloVe.

Table 5 compares the performance of different DL models using FastText and GloVe embeddings. When using FastText, CNN performs the weakest, with an *F*_1_-score of 0.72, while BiLSTM performs significantly better at 0.91. However, models trained with GloVe embeddings outperformed those trained with FastText. The CNN model with GloVe achieved the highest performance across all metrics (0.94), followed closely by BiLSTM with 0.93. This suggests that GloVe embeddings provide richer semantic representations for this task, leading to better model performance, especially for CNN. Overall, GloVe-based models outperformed their FastText counterparts, and CNN with GloVe achieved the best results.

**Table 5.** Results for deep learning models.

Models	Precision	Recall	*F*_1_-score	Accuracy
FastText: convolutional neural network	0.72	0.72	0.72	0.72
FastText: bidirectional long short-term memory	0.91	0.91	0.91	0.91
Global Vectors for Word Representation: convolutional neural network	0.94	0.94	0.94	0.94
Global Vectors for Word Representation: bidirectional long short-term memory	0.93	0.93	0.93	0.93

### Transformer Results

Multimedia Appendix 4 presents the performance comparison of 3 transformer-based models—RoBERTa-base, crosslingual language model (XLM)–RoBERTa-base, and BERT-base-uncased—across 4 key metrics: precision, recall, *F*_1_-score, and accuracy. The RoBERTa-base model (blue bars) consistently outperformed the others, achieving a score of 0.94 in all metrics. The XLM-RoBERTa-base model (red bars) performed equally well in recall and accuracy but lagged slightly in precision and *F*_1_-score. Meanwhile, BERT-base-uncased (green bars) had the lowest performance, with a score of 0.93 across all metrics. Although the differences are small, they highlight how model architecture influences classification performance, with RoBERTa-based models proving to be slightly more effective in this particular task.

Overall, RoBERTa-base outperformed the other models with the highest scores across all metrics, making it the most effective for this task. Although XLM-RoBERTa-base was close, BERT-base-uncased showed slightly lower performance.

### LLM Results

LLMs have revolutionized the field of artificial intelligence by enabling machines to understand and generate human-like text with remarkable accuracy. LLM models are trained on a large volume of textual data, allowing them to capture hidden patterns in language, comprehend complex queries, and produce coherent and contextually relevant responses. By using the capabilities of LLMs such as GPT-3.5 Turbo, researchers and developers can unlock innovative solutions, bridging the gap between human communication and machine intelligence. To attain this objective, we have used the power of OpenAI’s model for the sentiment analysis task and we evaluated its effectiveness using 4 metrics: precision, recall, accuracy, and *F*_1_-score. Multimedia Appendix 5 presents the performance of GPT-3.5 Turbo across the 4 key metrics, all achieving an impressive 0.95. This indicates that GPT-3.5 Turbo performs exceptionally well in classification tasks, likely benefiting from its large-scale pretraining and contextual understanding. Compared to traditional ML models or even DL architectures, its high and balanced performance across all metrics suggests strong generalization and robustness in text classification.

Overall, GPT-3.5 Turbo excelled, with a perfect balance across all metrics (0.95), making it a highly effective choice for text classification tasks.

Table 6 shows the class-wise performance metrics of our proposed methodology (GPT-3.5 Turbo) on 6 distinct classes, capturing both positive experiences (ie, pain relief, euphoria, relaxation) and negative outcomes (ie, nausea, sadness, respiratory depression), and highlights precision, recall, *F*_1_-score, and support (number of instances per class). Among the classes, euphoria, nausea, and respiratory depression showed the highest performance, achieving nearly perfect scores across all metrics. Euphoria, relaxation, and pain relief also performed well, with slight variations in precision and recall. Sadness, however, had the lowest recall (0.85) and *F*_1_-score (0.89), indicating that the model struggled slightly with detecting this class.

**Table 6.** Class-wise score for the GPT-3.5 Turbo model.

Class	Precision	Recall	*F*_1_-score	Support
Euphoria	0.97	0.97	0.97	588
Nausea	0.99	0.99	0.99	601
Pain relief	0.92	0.93	0.92	645
Relaxation	0.92	0.97	0.95	638
Respiratory depression	0.98	1	0.99	628
Sadness	0.94	0.85	0.89	643

Overall, the model performed exceptionally well across most classes, with nausea and respiratory depression achieving near-perfect classification. However, sadness had the lowest recall, suggesting room for improvement in detecting this category.

### Error Analysis

Multimedia Appendix 6 presents the top-performing models across various learning approaches based on their precision, recall, accuracy, and *F*_1_-score metrics. Among ML techniques, the XGBoost model excelled, with solid precision, recall, *F*_1_-score, and accuracy values of 0.92. In DL, the CNN model with GloVe embeddings achieved 0.94 in all metrics. For transfer learning, the roBERTa-base model matched this, achieving a score of 0.94 across the board as well. Finally, GPT-3.5 Turbo (an LLM) took the lead with slightly higher performance, boasting a precision, recall, *F*_1_-score, and accuracy of 0.95, showing its exceptional ability in handling complex tasks. Overall, each approach demonstrated strong performance, but GPT-3.5 Turbo stood out as the highest achiever.

Although RoBERTa-base achieved solid performance with an accuracy, precision, recall, and *F*_1_-score of 0.94, GPT-3.5 Turbo outperformed it with 0.95 across all metrics. This 1.06% performance improvement shows GPT-3.5’s superior ability to capture complex, nuanced language patterns and generalize better to diverse user sentiments related to opioid use. Although RoBERTa excels in domain-specific tasks, GPT-3.5 Turbo’s versatility allows it to handle a wider range of emotional expressions more effectively. As the dataset size increases, GPT-3.5 Turbo’s performance is expected to improve further, reinforcing its edge in predicting overdose risks and understanding nuanced user experiences.

**Figure 3.**
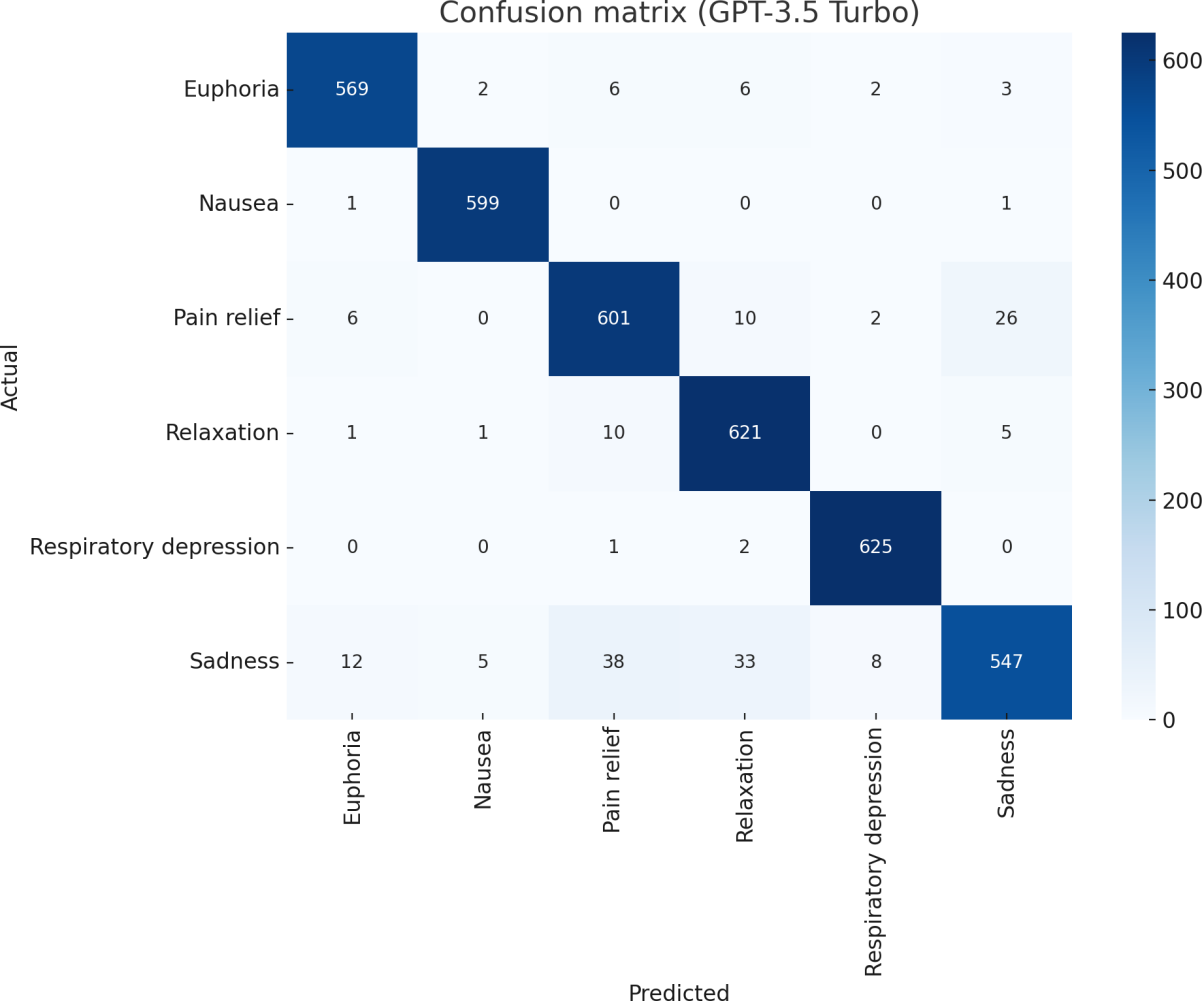
Confusion matrix of the proposed GPT-3.5 Turbo model.

Table 3 summarizes the learning approaches, models, and hyperparameters used across various ML and DL techniques. GPT-3.5 Turbo was fine-tuned with a learning rate of 2, 3 epochs, a batch size of 29, and seed=1,414,121,048, ensuring effective adaptation. Transformer models such as BERT, RoBERTa, and XLM-RoBERTa used a learning rate of 2e-5, 3 epochs, a batch size of 32, AdamW as the optimizer, and CrossEntropyLoss for classification tasks. ML models included KNN (n_neighbors=5, weights=‘uniform’), XGBoost (n_estimators=100, max_depth=6, learning_rate=0.3), DT (random_state=42, max_depth=10), and LR (random_state=42, max_iter=1000, C=0.1, solver=‘liblinear’). DL models like BiLSTM and CNN were trained with a learning rate of 0.1, 3 epochs, an embedding dimension of 300, and a batch size of 32. Each model’s hyperparameters were fine-tuned to optimize performance for specific tasks, ensuring efficient learning and improved accuracy. Figure 3 shows the confusion matrix of the proposed model (GPT-3.5 Turbo).

## Discussion

### Principal Findings

This study highlights the effectiveness of sentiment analysis in extracting meaningful insights from self-reported experiences with opioid drugs mixed with illicit substances. By leveraging YouTube comments as a data source, we were able to analyze public discourse on opioid use, uncovering both positive and negative experiences. Our classification system, comprising 6 sentiment-based categories, provided a structured approach to understanding the emotional and physical effects associated with opioid consumption. Notably, this method allowed us to identify key adverse effects such as nausea, respiratory depression, and sadness, alongside reported benefits like pain relief and euphoria.

A significant contribution of this research is the application of OpenAI models such as GPT-3.5 Turbo for sentiment analysis. The model achieved an *F*_1_-score of 0.95 in a multiclass setup, outperforming the baseline XGBoost model by 3.26%. This improvement underscores the utility of advanced NLP techniques in analyzing complex, health-related discussions. By automating the classification process, our approach reduces reliance on manual annotation and offers a scalable solution for monitoring opioid misuse trends. Such insights can enhance pharmacovigilance efforts, enabling real-time analysis of user-generated content to support public health initiatives.

### Limitations

Despite its contributions, this study has several limitations. First, the reliance on YouTube as the primary data source may not fully capture the diversity of opioid-related discussions across different social media platforms. Platforms such as X, Facebook, and Reddit have distinct user demographics and language patterns, which could influence sentiment classification outcomes. Expanding data collection to multiple platforms would improve the generalizability of our findings.

Second, the manual annotation process, while aimed at ensuring accuracy, remains inherently subjective. Variability in human interpretation of comments may introduce inconsistencies in the dataset. Future studies could explore semisupervised learning techniques or crowd-sourced annotations to enhance labeling reliability.

Additionally, the 6-class sentiment framework, while comprehensive, may not capture the full spectrum of opioid-related experiences. Refining the classification system to include more granular sentiment categories could provide deeper insights. Moreover, GPT-3.5 Turbo, despite its strong performance, exhibits occasional errors in interpreting medical terms and context-specific nuances, which may impact classification accuracy.

### Conclusions and Future Work

This study demonstrates the effectiveness of ML, DL, and LLMs in analyzing public sentiment surrounding opioid use mixed with other substances. By manually annotating YouTube comments into 6 distinct sentiment-based classes—capturing both positive effects (eg, pain relief, euphoria, relaxation) and negative experiences (eg, nausea, sadness, respiratory depression)—we provided a nuanced understanding of opioid-related discussions.

Our proposed methodology, using GPT-3.5 Turbo, achieved the highest *F*_1_-score of 0.95, outperforming traditional ML models such as XGBoost, which demonstrated an *F*_1_-score of 0.92. This significant improvement underscores the potential of LLMs in accurately identifying high-risk opioid use patterns from user-generated content.

By leveraging social media as a real-time source of self-reported experiences, this approach offers a scalable and less invasive method for opioid surveillance. The findings highlight the potential for artificial intelligence–driven tools to enhance health care interventions and public health strategies by identifying overdose risk more accurately. Future research can expand on this work by incorporating real-time monitoring, larger datasets, and additional language models to further improve predictive performance and intervention strategies.

In future work, we will focus on several key areas. First, we will expand the dataset to include comments from multiple social media platforms, such as Reddit, X, and Facebook, which will enhance the robustness and applicability of the model. Additionally, we plan to expand our dataset to include multilingual content to capture a broader spectrum of experiences across different language groups. Incorporating demographic and geographic metadata could further refine the analysis, providing insights into regional and population-specific trends in opioid use.

Second, refining the classification system by incorporating additional sentiment categories or leveraging hierarchical classification techniques could improve the granularity of sentiment detection. Finally, integrating real-time monitoring capabilities into public health frameworks could facilitate proactive intervention strategies. By developing automated tools for detecting emerging opioid-related trends, policymakers and health care professionals could respond more swiftly to potential risks, ultimately contributing to more effective opioid crisis management.

Overall, this research underscores the potential of sentiment analysis in public health surveillance and emphasizes the need for ongoing advancements in NLP methodologies to improve opioid misuse detection and intervention strategies.

## Supplementary material

10.2196/70525 Multimedia Appendix 1 Word cloud of keywords extracted from the dataset.

10.2196/70525 Multimedia Appendix 2 Class-wise label distribution in the dataset.

10.2196/70525 Multimedia Appendix 3 Statistical overview of the dataset.

10.2196/70525 Multimedia Appendix 4 Transformer results.

10.2196/70525 Multimedia Appendix 5 Performance metrics (precision, recall, and F1-score) for GPT-3.5 Turbo in the sentiment analysis task.

10.2196/70525 Multimedia Appendix 6 Top-performing models across various learning approaches based on their precision, recall, accuracy, and F1-score metrics.

## Abbreviations

BERT: bidirectional encoder representations from transformers
BiLSTM: bidirectional long short-term memory
CNN: convolutional neural network
DL: deep learning
DT: decision tree
GloVe: Global Vectors for Word Representation
KNN: k-nearest neighbors
LLM: large language model
LR: logistic regression
ML: machine learning
NLP: natural language processing
SVM: support vector machine
TF-IDF: term frequency-inverse document frequency
XGBoost: extreme gradient boosting
XLM: crosslingual language model
