# Supplementary figures and images for: Sentiment Analysis Using a Large Language Model–Based Approach to Detect Opioids Mixed With Other Substances Via Social Media: Method Development and Validation
Source: JMIR Infodemiology. 2025 Jun 19;5:e70525. doi: 10.2196/70525 (PMC12199843; doi:10.2196/70525)

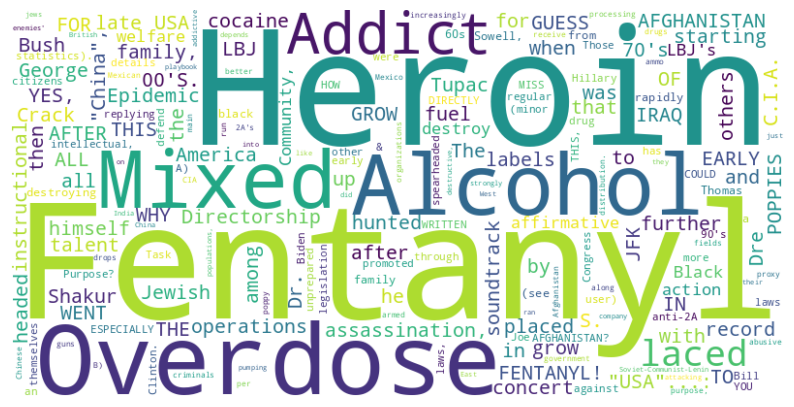

Supplement: Multimedia Appendix 1 [file infodemiology-v5-e70525-s001.png]

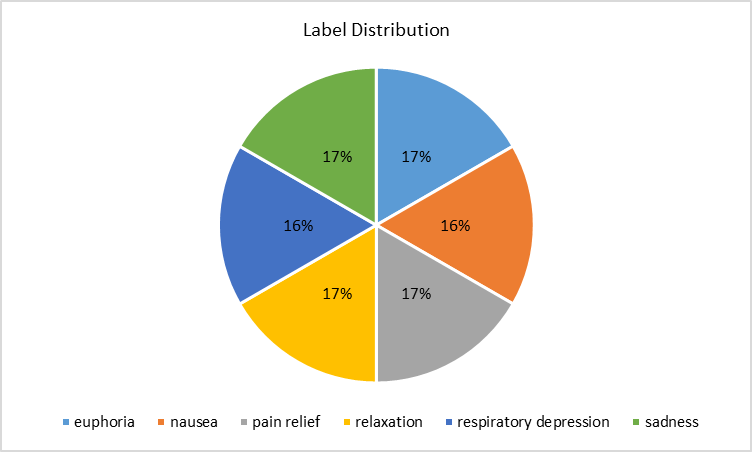

Supplement: Multimedia Appendix 2 [file infodemiology-v5-e70525-s002.png]

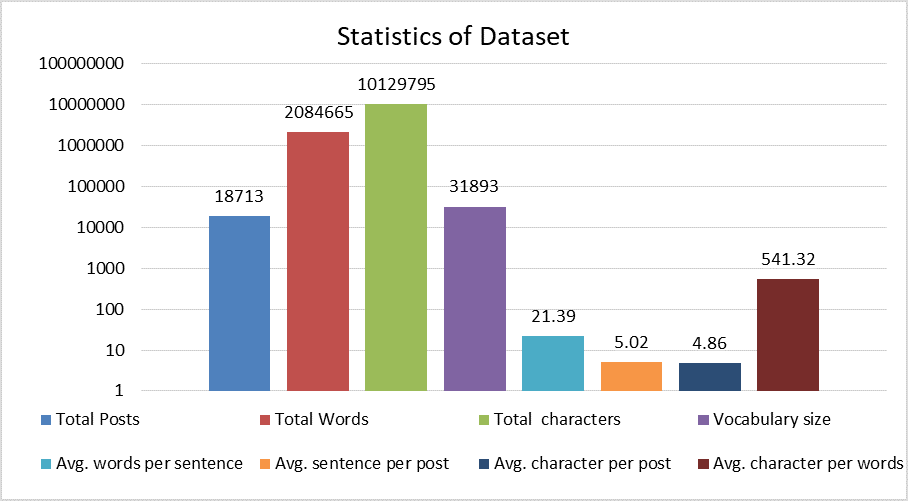

Supplement: Multimedia Appendix 3 [file infodemiology-v5-e70525-s003.png]

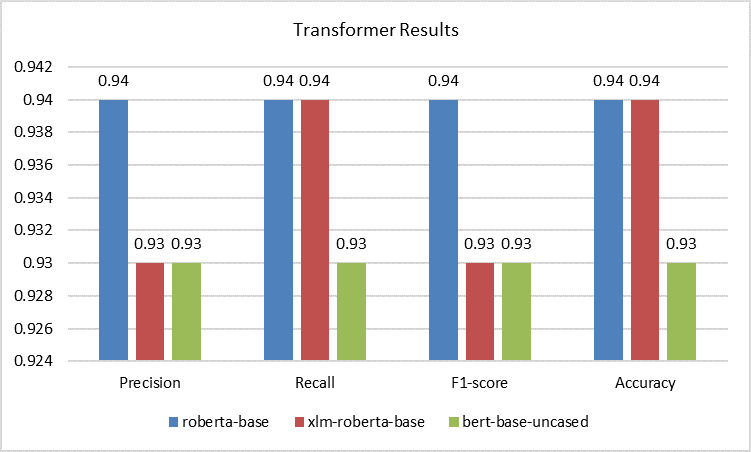

Supplement: Multimedia Appendix 4 [file infodemiology-v5-e70525-s004.png]

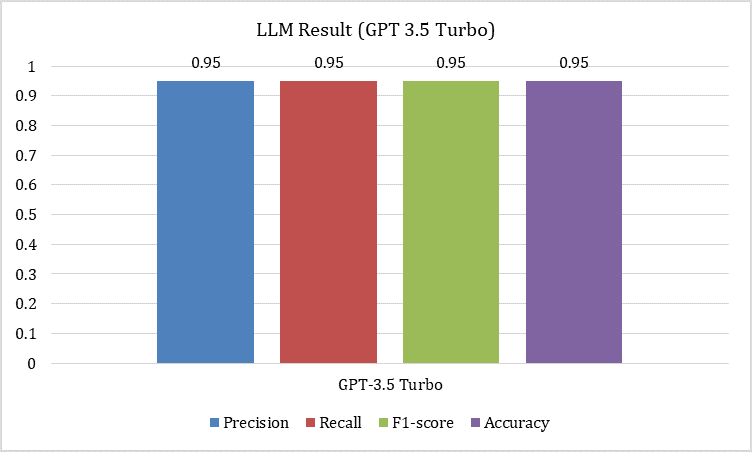

Supplement: Multimedia Appendix 5 [file infodemiology-v5-e70525-s005.png]

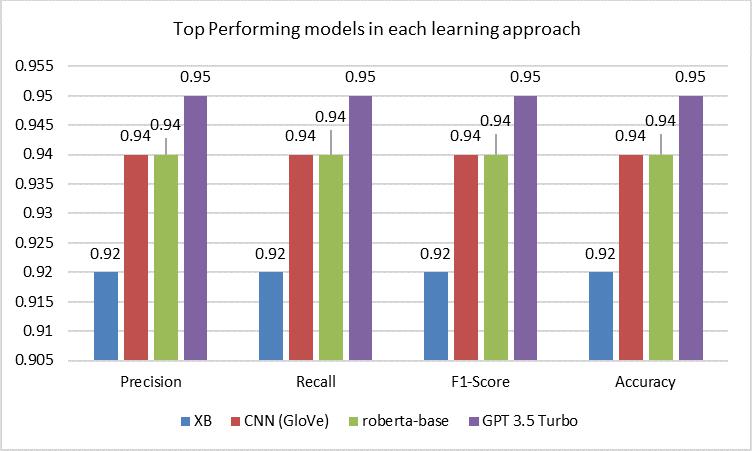

Supplement: Multimedia Appendix 6 [file infodemiology-v5-e70525-s006.jpeg]
